# Rapid On-Field Monitoring for Odor-Active Homologous Aliphatic Aldehydes and Ketones from Hot-Mix Asphalt Emission via Dynamic-SPME Air Sampling with Online Gas Chromatographic Analysis

**DOI:** 10.3390/molecules30173545

**Published:** 2025-08-29

**Authors:** Stefano Dugheri, Giovanni Cappelli, Ilaria Rapi, Riccardo Gori, Lorenzo Venturini, Niccolò Fanfani, Chiara Vita, Fabio Cioni, Ettore Guerriero, Domenico Cipriano, Gian Luca Bartolucci, Luca Di Giampaolo, Mieczyslaw Sajewicz, Veronica Traversini, Nicola Mucci, Antonio Baldassarre

**Affiliations:** 1 Department of Life Science, Health, and Health Professions, Link Campus University, 00165 Rome, Italy; 2 Department of Experimental and Clinical Medicine, University of Florence, 50134 Florence, Italy; giovanni.cappelli@unifi.it (G.C.); ilaria.rapi@unifi.it (I.R.); lorenzo.venturini@unifi.it (L.V.); niccolo.fanfani@unifi.it (N.F.); veronica.traversini@unifi.it (V.T.); nicola.mucci@unifi.it (N.M.); antonio.baldassarre@unifi.it (A.B.); 3 Department of Civil and Environmental Engineering, University of Florence, 50139 Florence, Italy; riccardo.gori@unifi.it; 4 QuMAP-PIN Foundation—Prato Campus of the University of Florence, 59100 Prato, Italy; chiara.vita@pin.unifi.it; 5 Regional Agency for Environmental Protection of Tuscany, 50144 Florence, Italy; f.cioni@arpat.toscana.it; 6 National Research Council of Italy—Institute on Atmospheric Pollution (CNR-IIA), 00010 Montelibretti, Italy; ettore.guerriero@cnr.it; 7 RSE SpA—Energy System Research, 20134 Milan, Italy; domenico.cipriano@rse-web.it; 8 Department of Neuroscience, Psychology, Drug Area and Child Health (NEUROFARBA), University of Florence, 50139 Florence, Italy; gianluca.bartolucci@unifi.it; 9 Department of Innovative Technologies in Medicine and Dentistry, University of “G. D’Annunzio” Chieti, 66013 Chieti, Italy; luca.digiampaolo@unich.it; 10 Institute of Chemistry, University of Silesia, 40006 Katowice, Poland; mieczyslaw.sajewicz@us.edu.pl; 11 Division of Occupational Medicine, Careggi University Hospital, 50139 Florence, Italy

**Keywords:** hot-mix asphalt, odor, SPME, PFBHA

## Abstract

Odorous emissions from hot-mix asphalt (HMA) plants are a growing environmental concern, particularly due to airborne aldehydes and ketones, which have low odor thresholds and a strong sensory impact. This study presents a field-ready analytical method for monitoring odor-active volatile compounds. The system uses dynamic solid-phase microextraction (SPME and SPME Arrow) with on-fiber derivatization via O-(2,3,4,5,6-pentafluorobenzyl)hydroxylamine (PFBHA) and is coupled to gas chromatography–mass spectrometry (GC–MS) for direct detection. A flow-cell sampling unit enables the real-time capture of aliphatic aldehydes and ketones under transient emission conditions. Calibration using permeation tubes demonstrated sensitivity (limits of detection (LODs) below 0.13 μg/m^3^), recovery above 85% and consistent reproducibility. Compound identity was confirmed using retention indices and fragmentation patterns. Uncertainty assessment followed ISO GUM (Guide to the Expression of Uncertainty in Measurement) standards, thereby validating the method’s environmental applicability. Field deployment 200 m from an HMA facility identified measurable concentrations that aligned with CALPUFF model predictions. The method’s dual-isomer resolution and 10 min runtime make it ideal for responding to time-sensitive odor complaints. Overall, this approach supports regulatory efforts by enabling high-throughput on-site chemical monitoring and improving source attribution in cases of odor nuisance.

## 1. Introduction

The exposure of people to chemical substances without their prior informed consent is a human rights issue [1]. Volatile organic compounds (VOCs) are emitted from a variety of sources. Interest in them is increasing due to the consistent number of VOCs emitted from different anthropogenic processes and their impact on health. In addition, VOC emissions can produce odor nuisance, affecting quality of life. In the last few years, an increasing number of complaints have been made by residents reporting odor nuisances [2,3]. Asphalt mixture plants are common sources of odors [4,5], but the characterization of their odor impact is challenging due to the plant configuration: some phases of their production processes involve large and open areas, and transient emissions are frequent and common.

The proposed analytical methods [6,7,8,9,10] were used to determine the odor-active compounds released by hot-mix asphalt (HMA), and showed that the greatest response was from homologous aliphatic aldehydes and ketones that contain no carbon (C)/carbon unsaturation—especially C4-C11 saturated fatty aldehydes (Table 1).

Volatile compounds with low odor detection threshold values (ODTVs) exert a substantial influence on perceived olfactory discomfort across varying intensities and hedonic valences, exhibiting an inverse correlation between threshold and molecular weight in the 120–130 Da range; moreover, saturated fatty aldehydes below 150 Da are predominantly malodorous, whereas higher-molecular-weight analogs tend to have a sweet or fruity aroma [15,16].

The analytical detection of carbonyl groups is performed directly on the emissions from the matrix. However, their detection according to the population that perceives the odor is not reported, causing a need for extremely sensitive and rapidly responding analytical methods capable of anticipating the perception of the odor.

Several European states (e.g., Portugal, Greece, Austria) currently lack dedicated odor legislation, and Germany limits regulatory measures to waste management activities. Italy, by contrast, enforces odor nuisance offenses under Penal Code article 674 [17] and, via Directorial Decree n. 309-MinAmbiente of 28 June 2023 (introducing article 272-bis) [18], authorizes the regional regulation of odor impact—explicitly including HMA production among regulated sources—and requires odor emissions to be described and evaluated using dynamic olfactometry, notwithstanding inter-individual sensitivity variability and the general absence of acceptability thresholds in dispersion models.

Dynamic olfactometry—although it represents the reference methodology EN 13725, 2003 [19]—presents some limitations, such as the subjectivity of the data (e.g., the lack of harmonization in materials, procedures or limits), the lack of definition of a smell fingerprint, and the impossibility of continuous monitoring. Odor concentration (OC) measurement accurately reflects the human detection or perception threshold, but cannot identify the odorants responsible for odor nuisances. This makes it impossible to attribute responsibility for the nuisance to a specific source, particularly in contexts where multiple anthropogenic activities with potential odor emissions are concurrently present within the affected area.

Thus, the use of short-time sampling with very high specificity and sensitivity to calculate the relationship between the concentration of the odorous compounds and the relative OC is encouraged. The use of analytical methods that are not affected by human bias response [20] is strongly recommended. In fact, compounds in the gas mixture can contribute to the odor stimuli to different extents, depending not only on their abundance in terms of composition, but also on their ODTVs and chemical and physiological interactions [21]. The ODTV is inversely proportional to odor, so it can be used as an index of the sensory impact of each volatile compound to the overall odorous compounds. Different ratios can be computed as an alternative approach that allows us to relate the chemical concentrations of the compounds to their odorous potential [22], such as the odor activity value (OAV) [23,24,25,26,27,28,29]. The OAV is defined as the ratio of a compound’s chemical concentration to its corresponding ODTVs. Understanding the link between OAV and OC can then be of crucial importance to simplify odor monitoring through analytical techniques.

In recent years, HMA emissions have been investigated in terms of chemical composition. In particular, various analytical techniques, including GC/MS (both dynamic headspace (HS) and microextraction) and GC-FTIR, have been employed to characterize asphalt emissions [30,31] and have consistently identified VOCs, SVOCs, and PAHs such as naphthalene, fluorene, phenanthrene, anthracene, and their derivatives. Moreover, these studies have highlighted the influence of emission temperature, the quantification of toxic compounds at ng/L–ng/m^3^ levels, and the detection of over 200 volatile species in modified asphalt mixtures. Nonetheless, only a few studies deal with the impact of VOCs and SVOCs when identifying the main compounds during odor nuisances [7].

To accurately assess external exposures to trace-level contaminants, novel methodologies—such as geospatial mapping, remote sensing, smartphone-based exposure sensors, and miniaturized high-throughput sampling—are imperative. In particular, on-site continuous sampling and analysis embody a sustainable analytical paradigm by eliminating sample transport and markedly reducing response times, thereby enhancing the real-time monitoring of environmental chemical stressors [32].

Solid-phase microextraction (SPME) is a sampling technique that has revolutionized gas chromatography (GC) by uniting sampling, enrichment, and injection in a single, miniaturized step [33]. Subsequent advances have further optimized its performance, including Fast Fit Assemblies (2009) for full automation and enhanced stability and the SPME Arrow (2015) with increased phase volume and mechanical robustness [34,35].

The use of SPME has been proposed for the measurement of various substances dispersed in air [36,37]. It can be used for both short (from 10 s to 15 min) and long sampling periods [38], and is suitable for on-site analytical applications. Some authors have already used O-(2,3,4,5,6-pentafluorobenzyl)hydroxylamine (PFBHA) hydrochloride as an on-fiber derivatizing agent with SPME to measure the levels of aldehydes in air using different GC detectors [39,40,41].

Moreover, by integrating a flow cell to facilitate automated, online volatile sampling from a continuous gas stream [42], on-fiber PFBHA derivatization with SPME—traditionally confined to static HS aldehyde analysis—can be extended to dynamic airborne monitoring when coupled to an ambient-interfaced microchamber.

In the last decade, mobile environmental laboratories, often mounted on vans, have played a pivotal role in the on-site sampling and analysis of ambient air, particularly in contexts involving transient odor nuisance and hazardous chemical emissions [43]. Their mobility allows for targeted investigations near suspected emission sources, enhancing responsiveness and spatial coverage. Recent studies emphasize the integration of electronic noses and GC systems to detect and quantify malodorous substances with high temporal resolution [44]. Moreover, comparative reviews highlight the strengths of mobile labs in assessing odor thresholds and pollutant dispersion in complex terrains [45].

In this study, we have developed a continuous, on-site air monitoring methodology coupled with gas chromatographic–mass spectrometric (GC–MS) analysis for the detection of sub-ppb concentrations of odor-active, homologous aliphatic aldehydes and ketones (C_4_–C_11_). The system is based on online flow cell sampling combined with both SPME and SPME Arrow techniques, loaded with PFBHA as the derivatizing agent. Given the trace-level concentrations targeted and the inherent complexity of on-site measurements, an evaluation of measurement uncertainty is integral to our approach. This analytical approach is proposed for implementation in receptor-based monitoring campaigns conducted in HMA production facilities, with the objective of identifying specific odor nuisances: the analytical methodology was implemented in situ at a distance of 200 m from a point-source emission to assess the presence of the target odorant compounds.

## 2. Results and Discussion

### 2.1. Analytical Performances

**Figure 1:**
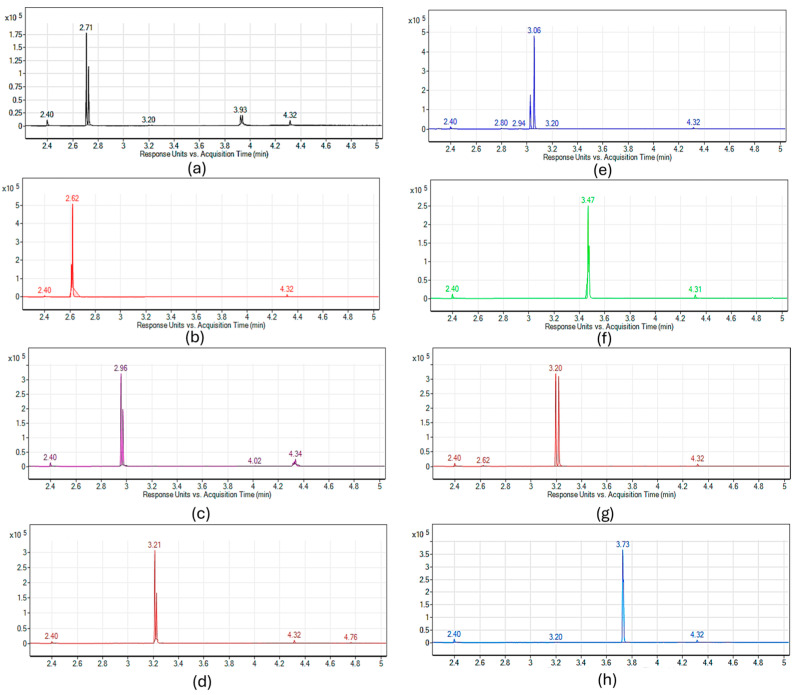
Chromatograms of aldehydes and ketones oxime obtained via SPME Arrow analytical set-up. (**a**) Butanal, (**b**) butan-2-one, (**c**) pentanal, (**d**) hexanal, (**e**) hexan-2-one, (**f**) heptanal, (**g**) heptan-3-one, (**h**) octanal.

The chromatograms of each aldehyde and ketone, obtained under the experimental conditions described in Section 3.2, are presented in Figure 1, while the monitored ions for each compound are listed in Table 2.

Given the use of PFBHA as a derivatizing reagent, the base peak for each resulting oxime corresponds to *m*/*z* = 181, which represents the characteristic fragment of the PFBHA moiety. Consequently, *m*/*z* = 181 was selected as the qualitative ion for all compounds. Additionally, a second ion was chosen for each carbonyl compound to enable quantification. Although this ion is shared among some analytes, the distinct retention times observed ensured the accurate identification of each individual substance. The results obtained from the Linear Temperature Programmed Retention Index (LTPRI) calculation, also shown in Table 2, reinforce the applicability of the method to discriminate between the carbonyl compounds surveyed.

The chromatograms also reveal that each carbonyl compound appears as a pair of peaks with varying relative intensities. This observation can be attributed to the stereochemical outcome of the reaction with PFBHA, which can statistically yield either the syn- or anti-oxime isomer. As these two products are diastereomers and can be resolved using the developed method, all subsequent data analyses were conducted by considering the combined contribution of both isomers [46].

Table 3 presents the analytical parameters for each investigated compound, calculated as described in the Analytical Performances section. The developed method demonstrates satisfactory performances in terms of sensitivity, reproducibility, and accuracy, in both SPME and SPME Arrow configurations. Regarding the calculation of the limit of detection (LOD), the use of indoor ambient air as a blank matrix was considered the most suitable compromise. Although the samples were diluted with medical air in the flow cell, indoor air better reflects real-life conditions, since the concentration of carbonyl compounds in indoor air is low but not zero; therefore, this approach can prevent the underestimation of the LOQ values. As far as the presence of syn- and anti-oximes are concerned, the most abundant peak for each analyte was considered to calculate the sensitivity of the method.

The performance of the developed method in terms of LOQ was compared to data in the existing literature. Only a few scientific papers have applied a similar analytical set-up for the determination of aldehyde and ketones using SPME or similar tools. Bourdin et al. [47], employing an instrumental configuration similar to the one proposed in this work, reported LOQ values for hexanal that were comparable to those obtained in this study. The use of SPME Arrow further reduced this value, yielding results similar to those achieved by the authors with a 20 min sampling time. However, addressing odor nuisance limits the feasibility of such extended sampling times (e.g., 40 min) [30], given the brief time window in which emissions may occur. This underscores the advantages of the specialized set-up developed in this work, where a 5 min sampling time was used. Moreover, the short on-site SPME, on-site sampling, and the GC-MS analysis allow for a more detailed spatial mapping of the odor nuisance, adding information about its chemical characterization and overcoming the qualitative evaluations given by dynamic olfactometry, electronic nose or similar direct reading instruments, such as PID [48].

The most abundant diastereoisomer was selected for the calculation of the LOD, as the developed method demonstrated sufficient sensitivity. In scenarios requiring the detection of lower concentrations, the method can be adapted by incorporating the combined signal of both derivatization products. Application of the method at an industrial facility (see Section 2.3) revealed consistent relative proportions of diastereoisomer formation across different emission sources, suggesting matrix independence. As expected, analyses conducted on different days—corresponding to different processed products—showed no significant variation in relative abundances, supporting the conclusion that the process is governed by the differential efficiency of derivatization. The implementation of rapid chromatographic runs in combination with pre-conditioned SPME and SPME Arrow fibers enables high-throughput analysis, with a total processing time of approximately 10 min from sampling to the generation of the analytical profile. This configuration fulfills the requirements for the effective monitoring of odor nuisances, considering the transient nature of such events.

### 2.2. Extended Measurement Uncertainty Assessment

Measurement uncertainty is defined as the parameter that characterizes the dispersion of values that could reasonably be attributed to the measurand, thereby establishing an interval within which the true value is expected to lie with a specified level of confidence. The Expanded Uncertainty is calculated by the multiplication of Combined Uncertainty (*U_c_*) with a coverage factor (K) according to the proposition of level of confidence. In general, the level of confidence for enormous datasets is considered at 95%, with more than 10 degrees of freedom [49] and thus K equal to 2.

The results of the evaluation of expanded uncertainty are reported in Table 4.

The data were obtained by the equation shown in Section 3.3.5. The expanded uncertainties were set and validated for our specific experimental conditions: the method repeatability was performed on five replicates of the calibration curve for each compound, concentration ranges from 1 to 10 µg/m^3^, and a sampling time of 5 min in the flow cell. The expanded uncertainties observed are relevant, considering the method application and the ODT values of the studied compounds. However, the performance is sufficient to observe exceedances of the ODT of each compound, even considering the uncertainty. Furthermore, there are no specific limits at which we must accept a result in terms of compliance or non-compliance with limit values, such as odor threshold or similar. In general, the decision rule that is currently widely used is that a result implies non-compliance with an upper or low limit if the measured value exceeds or is below the limit by the relative expanded uncertainty value [49].

### 2.3. On-Site Application: Emissions from a HMA Plant

No exceedance of the ODTs was observed for any compound analyzed during field analysis during the standard optimal operational conditions of the HMA plant at a distance of 200 m from the facility. Of the 30 samples collected, the last 3 in temporal sequence reported concentrations below the LOD for each compound, while the measured values for the remaining samples are summarized in Table 5.

**Figure 2:**
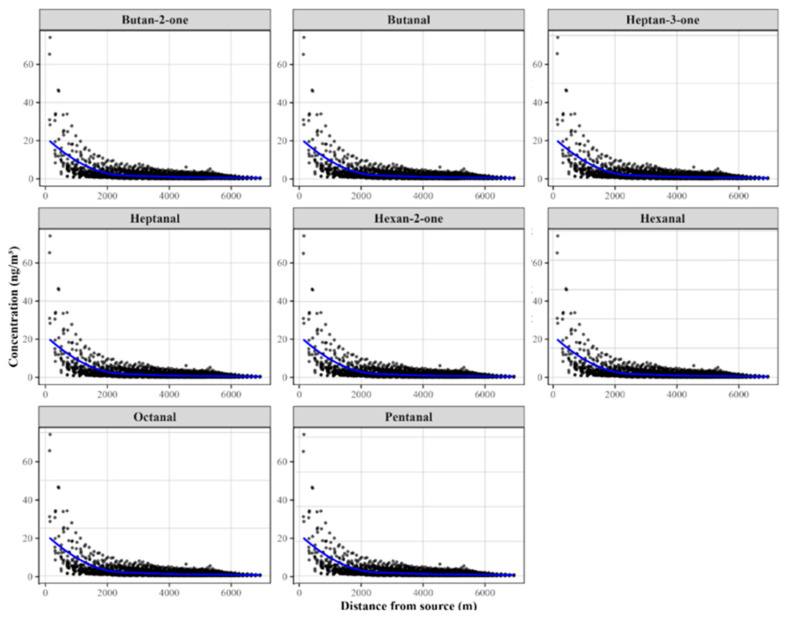
Graphs depicting the 98th percentile of hourly average receptor concentrations (ng/m^3^) estimated by CALPUFF, plotted against receptor distance from the emission source.

The field data corroborate, with reasonable approximation, the predictions obtained from the CALPUFF dispersion model (Figure 2); the stack emissions from the plant were modeled using the CALPUFF dispersion model to simulate aldehyde and ketone emissions, assuming a stack flow rate of 60,000 Nm^3^/h (Table 6).

Under controlled production conditions, stack emissions are unlikely to result in exceedances of the ODTs for aldehydes and ketones—compounds characteristic of the emission and responsible for its odorant fingerprint [4].

In many cases, even when industrial activities operate within regulatory limits, residents may report odor events not captured by traditional monitoring techniques [50]. The lack of real-time data, the transient nature of odor episodes, and limited legal thresholds across jurisdictions further complicate source attribution. Robust source-receptor modeling (e.g., via CALPUFF), community engagement, and innovative field-ready analytical tools are increasingly necessary to support transparent and defensible attribution in both scientific and legal contexts [51].

Mobile environmental laboratories equipped with GC–MS and electronic noses could enable the rapid, on-site assessment of odor nuisances by directly characterizing volatile, sulfur, and nitrogen compounds without the delays and losses inherent to sample transport [52]. Conti et al. demonstrated that mobile e-nose systems can continuously monitor hydrogen sulfide and ammonia emissions at livestock farms, correlating real-time sensor data with hedonic tone surveys to quantify community annoyance [53]. Field deployments near landfills using van-mounted GC–MS units have successfully speciated key malodorous components (e.g., dimethyl sulfide, mercaptans) and integrated meteorological data to refine dispersion models and delineate impact zones [54]. Moreover, participatory projects such as the Environmental Protection Agency (EPA)’s Odor Explore leverage mobile labs and citizen science apps to map odor events via smartphone-linked sensors, enhancing regulatory responsiveness and public engagement [43].

## 3. Materials and Methods

### 3.1. Standards and Reagents

Butanal (CAS 123-72-8), 2-butanone (CAS 78-93-3), pentanal (CAS 110-62-3), hexanal (CAS 66-25-1), 2-hexanone (CAS 591-78-6), heptanal (CAS 111-71-7), 3-heptanone (106-35-4), octanal (CAS 124-13-0), and O-(2,3,4,5,6-pentafluorobenzyl) hydroxylamine hydrochloride (PFBHA·HCl) (CAS 57981-02-9) were purchased from Sigma-Aldrich (Saint Louis, MO, USA). Polydimethylsiloxane/divinylbenzene (PDMS/DVB) 65 μm FFA-SPME (Cat. No. FFA57293-U) and 120 μm FFA-SPME Arrow (Cat. No. FFA27486) fibers were purchased from Chromline (Prato, Italy). MilliQ water 18 MΩ cm was obtained from Millipore’s simplicity system from Merck (Darmstadt, Germany), and further purified to eliminate aldehydes using the PURE UV3—4-Stage UV Water Purification System purchased from Pure n Natural Systems, Inc. (Steamwood, IL, USA). The gas helium (99.999%) was obtained from Air Liquid (Paris, France). HeadSpace screw-top 20 mL glass Vials (HSV) (Part No: 5188-2753) and Hdsp cap 18 mm magnetic PTFE/Sil (Part No.: 5188-2759) were purchased from Agilent Technologies (Santa Clara, CA, USA).

### 3.2. Procedure for Air Monitoring and Instrumental Configuration

**Figure 3:**
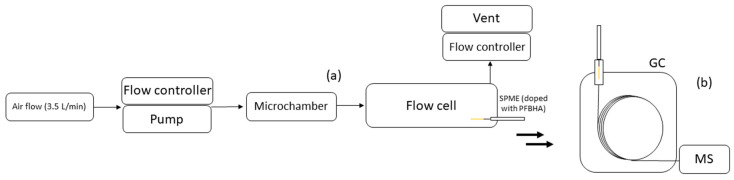
Schematic representation of air sampling via flow cell (**a**) and subsequent GC-MS analysis (**b**).

The routine on-fiber derivatization method is based on the procedure developed by Dugheri et al. [55]; SPME fibers were doped, exposing them under magnetic stirring (500 rpm) for 4 min in the HSV, containing 1 mL of a PFBHA carbonyl-free water solution (50 mg/mL), after an agitation step of 5 min at 60 °C. After PFBHA loading, the doped fiber was inserted for 5 min in the on-line flow cell coupled to the GC/MS set-up, modifying the one showed in Jian et al. [56]. Ambient air or a standard atmosphere was pumped into a microchamber at 3.5 L/min (±5% accuracy by a sampling pump); the chamber was connected to the flow-cell in which an airflow 3.5 L/min was pumped. The flow rate at the outlet of the flow-cell unit was verified by another flow meter (Defender 510 from Bios International Corp, Butler, US) at 3.5 L/min (±5% accuracy). The gas-phase aldehydes and ketones that passed through the flow-cell were then extracted by SPME and SPME Arrow pre-loaded with PFBHA. In this step, carbonyl compounds underwent a dehydration reaction with the derivatizing agent, and the SPME fiber was then thermally desorbed in the GC injection port for analytical separation and detection (Figure 3). An Agilent 7890 GC system (Santa Clara, USA) equipped with an Agilent 7000D triple quadrupole mass spectrometer and a split/splitless injector was employed. The injector was operated in split mode at 260 °C. Separation was achieved using a micro-bore Supelco SLB-5ms column (10 m × 100 μm I.D. × 0.1 μm film thickness; Merck KGaA, Darmstadt, Germany) with a helium carrier gas flow rate of 0.5 mL/min. The oven program began at 50 °C (1 min hold), followed by a temperature ramp of 75 °C/min to 90 °C, then 50 °C/min to 150 °C, 40 °C/min to 215 °C, 35 °C/min to 265 °C, and finally 30 °C/min to 320 °C, with a final hold of 1 min, resulting in a total run time of 8 min. Full automation of these procedures was achieved using a CTC PAL3 System xyz-Autosampler (CTC Analytics AG Industrie strasse 20 CH-4222, Zwingen, Switzerland) equipped with a Multi Fiber eXchange (MFX) system (Chromline, Prato, Italy), Liquid Syringe Tool (CTC Analytics AG, Zwingen, Switzerland) to guarantee an automated routine from the exchange of syringe, and FFA-SPME and FFA-SPME Arrow fibers for the injection. The flow-cell unit was installed on CTC systems on-line with GC. The instrumental setting was installed on a mobile lab installed in a high-roof long-wheelbase van fitted with a 5 kVA diesel generator, a 2 kW inverter, and a LiFePO_4_ battery bank recharged via a photovoltaic array. Laboratory ambient conditions were regulated by an independent HVAC unit with ±1 °C control and HEPA filtration, while vibration-damping mounts under the GC-MS and helium cylinders minimized mechanical disturbances. Continuous helium delivery was maintained by a dual-cylinder automatic switchover manifold equipped with moisture and hydrocarbon traps, guaranteeing the uninterrupted supply of 99.999% purity gas.

### 3.3. Method Performances

#### 3.3.1. Dynamic FA Atmosphere Standard by Permeation Tube

Calibration was performed with a dynamic calibration system for both aldehydes and ketones. Standard atmospheres were generated by means of permeation tube devices filled with the relative carbonyl compound, purchased from Fine Metrology (Spadafora, Messina, Italy). Each tube was calibrated according to the EPA [57]. A calibration gas generator with temperature controlling system (Sonimix 6000C1, LNI Swissgas, Versoix, Switzerland) was employed to generate C_4_–C_11_ fatty aliphatic carbonyl atmospheres at constant concentrations from one permeation tube. The temperature of the chamber, which directly affects the Permeation Rate (PR) of the carbonyls gas, was digitally controlled at 60 °C. According to the manufacturer, the desired volumetric concentration is established or changed by simply varying the inert carrier gas flow (which sweeps the calibration gas from the chamber) from 0.5 to 15 L/min, using a home-made dilution system [58]. The gas concentration generated from permeation tubes with different dilution gas flows can be represented by Equation (1):

(1) $${\left[C_{4}-C_{11}\right]}_{air}=\frac{W}{T}\;\times \;F_{air\;}^{-1},$$

where [*C*_4_ − *C*_11_]*_air_* is the concentration of each *C*_4_ − *C*_11_ in the air (µg/m^3^), *F_air_* is the airflow (L/min), and *W*/*T* is the PR (ng/min) given by W, the *C*_4_ − *C*_11_ weight loss (ng) and *T*, the measurement interval (min).

Calibration curves consisting of 5 levels were obtained by diluting the generated standard using 15, 10, 5, 2.5 and 1 L/min as outflow rate from the calibrator. This set-up allowed each of the carbonyl compounds to be generated in the concentration range 0.67 to 10 µg/m^3^.

#### 3.3.2. O-(Pentafluorobenzyl) Oxime-Derivate Recoveries

To evaluate the yield of the presented method for each investigated analyte, hexane solutions of the PFBHA derivatives were directly injected in the GC system and regression curves were constructed. In the Results section, the yields of derivatization are reported for each compound, evaluating two control gaseous standards at a concentration of 1 and 5 µg/m^3^, respectively, as the mean value obtained among the two measurements. These standards were prepared in triplicate, according to the procedure described in Section 3.3.1.

#### 3.3.3. Analytical Performances

The inter-day and intra-day performance of the method were assessed to evaluate its precision and reproducibility. Inter-day variability was determined over six non-consecutive days using three independent sets of calibration and standard solutions per day, from which daily average calibration curves were generated. Intra-day performances were evaluated by preparing and sequentially analyzing six distinct sets of calibration and standard solutions within a single analytical session.

Least squares linear regression was used to obtain the best-fitting function. Reliable *LOD* values were obtained using Equation (2):

(2) $$LOD=\frac{3.3\;\sigma_{b}}{a},$$

The standard deviation of the peak area obtained from six consecutive injections of a blank sample (i.e., ambient air) was used to estimate *σ_b_*, while a denotes the slope of the calibration curve for each analyte. The LOQ was defined as three times the *LOD* [59]. To assess method sensitivity, the signal-to-noise (S/N) ratio approach was preferred over the use of Y intercepts. This choice was motivated by the employment of medical air for the dilution of gaseous samples, allowing the use of a blank matrix more representative of the actual analytical conditions. Precision was evaluated by calculating the relative standard deviation (RSD) of quantitative results obtained from replicate analyses of the two control gaseous standards described in the O-(pentafluorobenzyl) oxime-derivate recoveries section. Accuracy was assessed by determining the recovery, expressed as the ratio between the measured and nominal concentrations of the control samples. Both parameters are expressed as the mean value among the two levels.

#### 3.3.4. Qualitative Analysis Identification of Aldehyde and Ketones by LTPRI

Retention indices represent a fundamental tool for the identification of volatile compounds in gas chromatography. Initially introduced by Kovats for isothermal separations [60], the concept was later adapted by van den Dool and Kratz [61] for use in linear temperature-programmed analyses. The latter approach, known as the *LTPRI* [34,37,62,63,64], is the most widely adopted. The *LTPRI* was calculated under the same chromatographic conditions applied to the sample, according to Equation (3):

(3) $$LTPRI=100\times \frac{t_{R(A)}-t_{R(C)}}{t_{R\left(C+1\right)}-t_{R(c)}}+100\times C,$$

where $t_{R(A)}$ represents the retention time of the analyte, $t_{R(C)}$ the retention time of the n-alkane eluting immediately prior to the analyte, $t_{R(C+1)}$ that of the n-alkane eluting immediately after, and *C* the number of carbon atoms in the preceding n-alkane. Volatile compounds were identified by comparing both their mass spectra with reference library data and their experimentally determined *LTPRI* values with literature or database standards.

#### 3.3.5. Extended Measurement Uncertainty Assessment

The UNI ISO ENV 13005:2000 [65] standard provides general guidance on expressing measurement uncertainty (often abbreviated to GUM, or Guide to the Expression of Uncertainty in Measurement). The “deconstructive” (or bottom-up) approach starts by estimating the contributions of individual sources of uncertainty, such as instruments, repeatability, the environment and the operator, and then combining these components into a compound (standard) uncertainty. The overall uncertainty associated with the final result of the analyte is expressed as Expanded Uncertainty (*U_e_*) at a certain level of confidence (e.g., 95%). The calculation procedure identifies the primary sources of uncertainties and quantifies their respective contributions to the total uncertainty of the final results.

The propagation of these individual uncertainties from different sources is expressed as combined relative uncertainty (*U_c_*), which is calculated using Formula (4):

(4) $$U_{c}=\sqrt{u^{2}\left(x\right)+u^{2}\left(y\right)\ldots \ldots }\times 10^{-2},$$

In the experimental settings, the following sources of uncertainty $u^{2}\left(x\right)$ are evaluated:

- Repeatability of the method;
- The calculation of the amount of aldehyde using the calibration curve;
- The angular coefficient a_2_ of aldehyde using the calibration curve;
- Sampling time;
- Concentration of aldehyde in the flow cell (stability of PR and total gas flow in the flow cell).

In modern concepts of chemical metrology, analytical chemists are increasingly required to make a statement on the level of uncertainty associated with the estimate of a measurand [66]. This comprehensive uncertainty assessment—encompassing contributions from sampling, extraction, calibration, and instrumental analysis—ensures the reliability, comparability, and overall interpretability of analytical results, and forms the cornerstone for validating the performance and acceptability of the method in real-world environmental monitoring applications.

### 3.4. On-Site Application: Emission from a HMA Plant

A sequence of 30 samplings was carried out on subsequent days using the developed analytical system under field conditions, with a sampling time of 5 min followed by a 2 min pause, at approximately 200 m from the stack of a HMA plant—corresponding to the location of the nearest receptors. The objective of this test was to evaluate whether the experimental values obtained through the developed analytical system, which was installed in a mobile laboratory to optimize operational efficiency, were comparable to the modeled data at receptor level.

## 4. Conclusions

Olfactory nuisances from anthropogenic activities remain a complex environmental challenge with significant social implications. Advancing standardized methodologies and fostering interdisciplinary approaches are essential for enhancing odor assessment. The rapid SPME Arrow–GC–MS protocol developed demonstrates that high-resolution chemical characterization of transient odor events can be achieved with a 5 min sampling window. Sensitivity, repeatability and linearity confirm that this method offers performance on par with, or superior to, longer extraction techniques, while the integration into a mobile laboratory platform enables on-site, near-real-time analysis. By combining abbreviated sampling times with field deployability, this method overcomes the spatial and temporal limitations inherent to single-point, qualitative odor assays such as dynamic olfactometry or standalone electronic noses. In practice, mobile SPME Arrow deployments can map the dispersion of odor plumes around hot mix asphalt plants (and other fugitive sources), capture emissions at the moment and location of receptor exposure, and provide the objective chemical profiles needed to attribute and prioritize odor complaints. Future work will focus on expanding the compound library and calibration protocols to cover a broader spectrum of odorants and embedding automated meteorological and dispersion models to predict and preempt community exposure.

## Figures and Tables

**Table 1:** Aldehydes and ketones representative of the hot-mix asphalt fingerprint.

Compound Name/Formula	CAS n.	MW * [11,12,13] Da	BP [11,13,14] °C	Diffusion Coefficient 10^−5^ m^2^/s **	VP [11,12,13] Pa	RVD *** [11] Air = 1	Odor Character [11,12,13,14]	Odor Threshold [14] µg/m^3^
Butanal/C4H8O	123-72-8	72	75	1.18	12,200	2.5	Pungent	1.7
2-Butanone/C4H8O	78-93-3	72	79	1.18	10,500	2.41	Mint	1300
Pentanal/C5H10O	110-62-3	86	103	1.08	3400	3.0	Acrid, pungent	1.4
Hexanal/C6H12O	66-25-1	100	129	1.0	1100	-	Strong, grass	1.15
2-Hexanone/C6H12O	591-78-6	100	126	1.0	360	3.5	Sharp	9.8
Heptanal/C7H14O	111-71-7	114	153	0.94	3500	-	Pungent, fatty	0.84
3-Heptanone/C7H14O	106-35-4	114	148	0.94	200	3.9	Powerful, fruity	0.47
Octanal/C8H16O	124-13-0	128	171	0.88	206	-	Pungent, citrus-like	0.05

* Molecular weight (MW); ** under standard ambient conditions (approximately 298 K and 1 atm); *** relative vapor density (RVD).

**Table 2:** O-(pentafluorobenzyl)oximes, derivatives of aldehydes and ketones with PFBHA: ions and LTPRI value for qualitative analysis.

Compound Name/Formula.	MW Da	EI/MS Ions	LTPRI ^a^
Butanal *O*-(pentafluorobenzyl)oxime	267	181 (100), 239 (24)	1285
2-Butanone *O*-(pentafluorobenzyl)oxime	267	181 (100), 250 (41)	1248
Pentanal *O*-(pentafluorobenzyl)oxime	281	181 (100), 239 (35)	1360
Hexanal *O*-(pentafluorobenzyl)oxime	295	181 (100), 239 (31)	1459
2-Hexanone *O*-(pentafluorobenzyl)oxime	295	181 (100), 57 (22)	1391
Heptanal *O*-(pentafluorobenzyl)oxime	309	181 (100), 239 (34)	1555
3-Heptanone *O*-(pentafluorobenzyl)oxime	309	181 (100), 253 (39)	1466
Octanal *O*-(pentafluorobenzyl)oxime	323	181 (100), 239 (36)	1650

^a^ LTPRI given for the first and second eluting isomer (without assignments for syn or anti configuration) on apolar (RI-5) separation columns.

**Table 3:** LOD, limits of quantification (LOQ), recovery, RSD, and accuracy obtained for GC-MS analysis in SIM mode with 5 min of sampling time.

Compound	LOD µg/m^3^		LOQ µg/m^3^		LOD µg/m^3^		LOQ µg/m^3^		Recovery %		RSD %		Accuracy %	
	Intra-Day		Intra-Day		Inter-Day		Inter-Day							
	(a)	(b)	(a)	(b)	(a)	(b)	(a)	(b)	(a)	(b)	(a)	(b)	(a)	(b)
Butanal	0.051	0.021	0.153	0.063	0.049	0.014	0.147	0.042	91	97	4.5	3.8	98	94
2-Butanone	0.061	0.031	0.183	0.093	0.068	0.037	0.204	0.111	89	87	3.6	3.1	94	92
Pentanal	0.059	0.024	0.177	0.072	0.063	0.026	0.189	0.078	87	86	3.2	2.6	90	97
Hexanal	0.062	0.021	0.186	0.063	0.051	0.034	0.153	0.102	88	97	2.5	2.0	92	89
2-Hexanone	0.085	0.025	0.255	0.075	0.070	0.019	0.210	0.057	93	96	2.2	2.4	89	96
Heptanal	0.079	0.027	0.237	0.081	0.061	0.035	0.183	0.105	94	94	4.1	2.9	88	99
3-Heptanone	0.127	0.041	0.381	0.123	0.119	0.027	0.357	0.081	90	99	3.6	3.0	94	91
Octanal	0.082	0.026	0.246	0.072	0.093	0.033	0.279	0.099	89	98	2.8	2.4	93	87

(a) SPME, (b) SPME Arrow.

**Table 4:** Results of the expanded uncertainty for aldehyde and ketones, obtained using SPME arrows, calibration curves with gaseous standards by permeation tubes, and a sampling time of 5 min.

Compound	*U_Cald_* (10^2^)	v_eff_	K_p = 0.95_	*U_e_*%
Butanal	17.8	>10	2	35.6
2-Butanone	21.2			42.4
Pentanal	16.5			33.0
Hexanal	14.3			28.6
2-Hexanone	20.1			40.2
Heptanal	19.3			38.6
3-Heptanone	17.9			35.8
Octanal	22.9			45.8

**Table 5:** On-site results of SPME Arrow 200 m from HMA facility.

Substance	Number of Samples	Min	Max
		µg/m^3^	
Butanal	30	<LOD (0.021)	0.083
2-Butanone		<LOD (0.031)	0.118
Pentanal		<LOD (0.024)	0.082
Hexanal		<LOD (0.021)	0.087
2-Hexanone		<LOD (0.025)	0.093
Heptanal		<LOD (0.027)	0.106
3-Heptanone		<LOD (0.041)	<0.041
Octanal		<LOD (0.026)	0.083

**Table 6:** Aldehyde and ketone concentration for the stack emissions of hot-mix asphalt plan.

	Butanal	Butan-2-One	Pentanal	Hexanal
µg/m^3^				
Average	227.62	117.39	129.97	153.01
Dev. Std.	113.91	58.58	69.28	85.23
Median	224.28	115.91	123.99	140.73
	**Hexan-2-One**	**Heptanal**	**Heptan-3-One**	**Octanal**
µg/m^3^				
Average	62.98	105.7	52.79	46.27
Dev. Std.	41.94	74.01	57.15	34.88
Median	45.08	73.04	19.46	42.1

## Data Availability

The information obtained in the study has been included in the article; no further data is available.

## Abbreviations

The following abbreviations are used in this manuscript:

HMA	Hot-Mix Asphalt;
SPME	Solid-Phase MicroExtraction;
PFBHA	O-(2,3,4,5,6-pentafluorobenzyl)hydroxylamine;
GC-MS	Gas Chromatography–Mass Spectrometry;
VOCs	Volatile Organic Compounds;
RVD	Relative Vapor Density;
ODTVs	Odor Detection Threshold Values;
LOD	Limits Of Detection;
LOQ	Limits of Quantification;
OC	Odor Concentration;
OAV	Odor Activity Value;
EPA	Environmental Protection Agency;
PR	Permeation Rate;
RSD	Relative Standard Deviation;
LTPRI	Linear Temperature Programmed Retention Index;
GUM	Guide to the Expression of Uncertainty in Measurement.
